# Exploring racial and ethnic diversity trajectories and diabetes prevalence in the United States

**DOI:** 10.1016/j.socscimed.2026.119205

**Published:** 2026-03-18

**Authors:** Jiue-An Yang, Calvin P. Tribby, Anna Dmowska, Qian Xiao, Marta M. Jankowska

**Affiliations:** a Population Sciences, Beckman Research Institute, City of Hope National Medical Center, Duarte, CA, USA; b Department of Geoinformation, Institute of Geoecology and Geoinformation, Adam Mickiewicz University, Poznań, Poland; c Department of Epidemiology, School of Public Health, The University of Texas Health Science Center at Houston, Houston, TX, USA

**Keywords:** Segregation, Structural racism, Spatial epidemiology, Neighborhood change, Chronic disease, Health disparities, Multilevel modeling

## Abstract

This study investigates whether long-term changes in neighborhood racial and ethnic diversity are associated with adult diabetes prevalence across the contiguous United States. We calculated diversity indexes for US census tracts (n = 72,033) using decennial census data from 1990 to 2020. Five diversity trajectory clusters were identified using K-means clustering. Diabetes prevalence in 2019 was obtained from the CDC’s PLACES dataset. Linear mixed models (LMMs) assessed global associations between diversity trajectories and diabetes prevalence, adjusting for age, sex, poverty, marital status, and public insurance. Geographically weighted regression (GWR) examined local variations in these associations. Compared to the reference group (low and stable diversity), tracts with increasing or high diversity had significantly lower diabetes prevalence. In fully adjusted LMMs, diversity trajectory clusters characterized by large increases or high diversity by 2020 showed the largest negative associations with diabetes prevalence (e.g., cluster 4: 0.71, 95% CI: 0.76 to −0.65). GWR revealed spatial heterogeneity in these relationships: tracts with significant negative associations were most concentrated in the South, but also appeared in urban areas of the Midwest and Northeast. Neighborhoods with increasing racial and ethnic diversity over time were associated with lower diabetes prevalence, independent of key socioeconomic factors. These findings suggest that historical trajectories of integration may shape present-day diabetes prevalence at the neighborhood level and highlight the value of incorporating demographic change into spatially aware public health strategies.

## 1. Introduction

Over the past four decades, diabetes prevalence in the United States has increased dramatically, roughly doubling from 1990 to the late 2000s before leveling off in the 2010s (Geiss et al., 2014; Wang et al., 2021). This growing burden has not been evenly distributed. Racial and ethnic minorities experience disproportionately high diabetes burden, with recent estimates indicating prevalence among Black and Hispanic/Latino adults nearly twice that of non-Hispanic whites (on the order of 11–12% vs. 6–7%) (Centers for Disease Control and Prevention, 2024a). These disparities have persisted or even widened over time, as diabetes incidence plateaued in white populations but continued to rise among Black and Hispanic groups into the 2000s (Menke et al., 2015). Geographic patterns mirror these inequities: the South consistently exhibits the highest diabetes prevalence of any US region, in contrast to lower rates in the Northeast and West. Several Southeastern states report adult diabetes prevalence well above 15%, roughly double the levels seen in parts of New England or the Mountain West (Gurka et al., 2018). A “diabetes belt” spanning 15 states in the South has been identified, encompassing counties with diabetes prevalence ≥11% and a concentration of high-risk factors (Barker et al., 2011).

Understanding the racial and ethnic makeup of a geographic area provides demographic context and a proxy measure for underlying social and economic conditions driven by societal and structural inequities, which in turn shape health outcomes. *Residential segregation* is defined as the degree to which two or more groups live separately from one another, in different parts of the urban environment (Massey and Denton, 1988). It is commonly measured with an index of dissimilarity; however other measures are also utilized (Massey and Denton, 1988). Racial residential segregation is posited as a fundamental cause of racial health disparities due to structural disadvantages including neighborhood disinvestment, limited economic opportunities, poorer educational outcomes, reduced healthcare opportunity, and greater hazardous environmental exposures (Gaskin et al., 2012; Osypuk and Acevedo-Garcia, 2010; Schwartz et al., 2022; Williams and Collins, 2001). *Racial diversity* refers to the relative balance or mix of racial and ethnic groups in a population, indicating the extent to which multiple groups are present. Racial diversity is commonly measured by entropy (or standardized entropy) and is described as racial heterogeneity of a population (White, 1983). Cities and neighborhoods with increased racial diversity may see higher social cohesion, greater resource distribution, and reduced poverty (Hipp and Kim, 2023;Wu et al., 2011). While racial diversity and residential segregation both describe how populations are distributed, they are distinct and often inversely related at the neighborhood level; higher diversity may imply lower segregation as groups mix rather than live apart (Holloway et al., 2012). Each of these contextual factors can influence health outcomes, particularly chronic conditions like diabetes, by shaping the environments and opportunities available to residents.

Studies specifically assessing associations between residential segregation and diabetes prevalence are mostly null (Kershaw and Pender, 2016). Three cross-sectional studies have assessed residential segregation associations with diabetes prevalence among black adults, with mixed findings. Two studies reported no association (Gaskin et al., 2014; Piccolo et al., 2015), but a recent study that connected electronic health records to an index of racial isolation (measuring the extent to which black residents are exposed to one another) found segregation to be associated with greater diabetes prevalence for both Black and white residents (Bravo et al., 2018). Separately, diabetes mortality rates were higher in more segregated neighborhoods, albeit with large variations between US cities (Hunt et al., 2014; Rosenstock et al., 2014). A study examining historic redlining practices in Seattle found that it explained between 51% and 60% of the variation in the neighborhood diabetes mortality rate (Linde et al., 2022). There are fewer studies on the associations between neighborhood-level racial diversity and diabetes. One study indicates that diabetes prevalence may be similar between racial/ethnic groups residing in census tracts with 35% Black and 35% white residents with similar socioeconomic status (LaVeist et al., 2008). Another study found that increased racial/ethnic diversity was associated with a decrease in metabolic syndrome (of which one component is high fasting blood sugar) (Li et al., 2019). Some explanations for the mixed and null findings are the use of different segregation measures, or some studies use proportion of a race/ethnic group, which is not a formal segregation measure (Oka, 2023). Additional limitations are that no single study may account for the complex relationships between neighborhood environment factors, such as food access, green space, housing, transportation, or social cohesion with diabetes prevalence (Mujahid et al., 2023). Finally, understanding drivers of diabetes disparities requires data that can compare prevalence between racial and ethnic groups, while addressing confounding between race/ethnicity, socioeconomic status, and residential segregation (LaVeist et al., 2008).

Despite growing interest in the role of neighborhood environments in shaping chronic disease risk, research explicitly examining racial diversity and diabetes remains scarce. Existing studies have largely assessed residential segregation or racial diversity at a single time point, limiting insight into the dynamic nature of demographic change. Although cross-sectional evidence suggests residential segregation may be associated with diabetes risk (Kershaw and Pender, 2016), prospective studies are few and yield mixed findings. One 25-year study found no association between neighborhood segregation and incident diabetes (Mayne et al., 2020), and studies of related constructs, such as social cohesion, report inconsistent results (Christine et al., 2015; Gebreab et al., 2017). Critically, no studies have investigated how trajectories of racial and ethnic diversity over time are associated with diabetes outcomes. This represents a significant gap, as understanding the impact of shifting demographic compositions may reveal mechanisms related to social integration, resource distribution, and structural disadvantage that static measures overlook. These neighborhood processes are particularly relevant for diabetes, a condition strongly shaped by place-based exposures including food access, opportunities for physical activity, environmental stressors, and access to preventive care. By capturing long-term changes in racial and ethnic composition, diversity trajectories may reflect evolving neighborhood conditions that influence cumulative diabetes risk.

Given the substantial demographic changes in the United States—where the White population declined from 75% in 1990 to 48% in 2020, and the Hispanic and Asian populations rose markedly during the same period—capturing these changes and understanding their potential health impacts is essential. Studies indicate a general trend of rapidly increasing diversity and a gradual reduction in multigroup segregation in metropolitan areas (Dmowska and Stepinski, 2023; Ellis et al., 2017; Frey, 2022; Johnson and Lichter, 2022; Walker, 2018). Frey (2022) found that between 2010 and 2020, all 56 major metropolitan areas he studied became more racially diverse, primarily due to stagnation or decline in White populations and the continued growth and dispersion of Latino/Hispanic and Asian American communities. Johnson and Lichter (2022) reported similar trends in rural areas, where increased racial and ethnic diversity resulted largely from decreasing White populations alongside growth among minority groups. Dmowska and Stepinski (2023) observed rapid increases in diversity and a gradual reduction of segregation in 61 major U.S. cities from 1990 to 2020. Notably, cities that started out more diverse and less segregated in 1990 saw slower rates of change, while those with lower initial diversity experienced more rapid diversification. Ellis et al. (2017) highlighted that racial diversity within neighborhoods across U.S. metropolitan areas saw a significant increase between 1990 and 2010. The study concluded that the rate of neighborhood diversity growth in the 2000s was substantially faster than predicted by trends from the 1990s. The implications of these evolving patterns for diabetes outcomes, particularly among Hispanic and Asian groups, remain largely unexplored and underscores the need for research that accounts for the temporal dynamics of racial diversity (Yang et al., 2020).

The goal of this study is to examine associations of racial diversity trajectories from 1990 to 2020 with model-based estimated diabetes prevalence in 2019 for the contiguous US at the census tract level. We first identify different groups of racial and ethnic diversity trajectories and examine these groups’ characteristics and spatial patterns. We then model the associations between diversity trajectory groups and model-based estimated diabetes prevalence. We hypothesize that areas with increasing diversity will be associated with lower model-based estimated diabetes prevalence. Finally, we examine how modeled associations vary spatially with a focus on urban/rural and regional differences.

## 2. Methods

### 2.1. Prevalence of model-based estimates of diagnosed diabetes

**Fig. 1.**
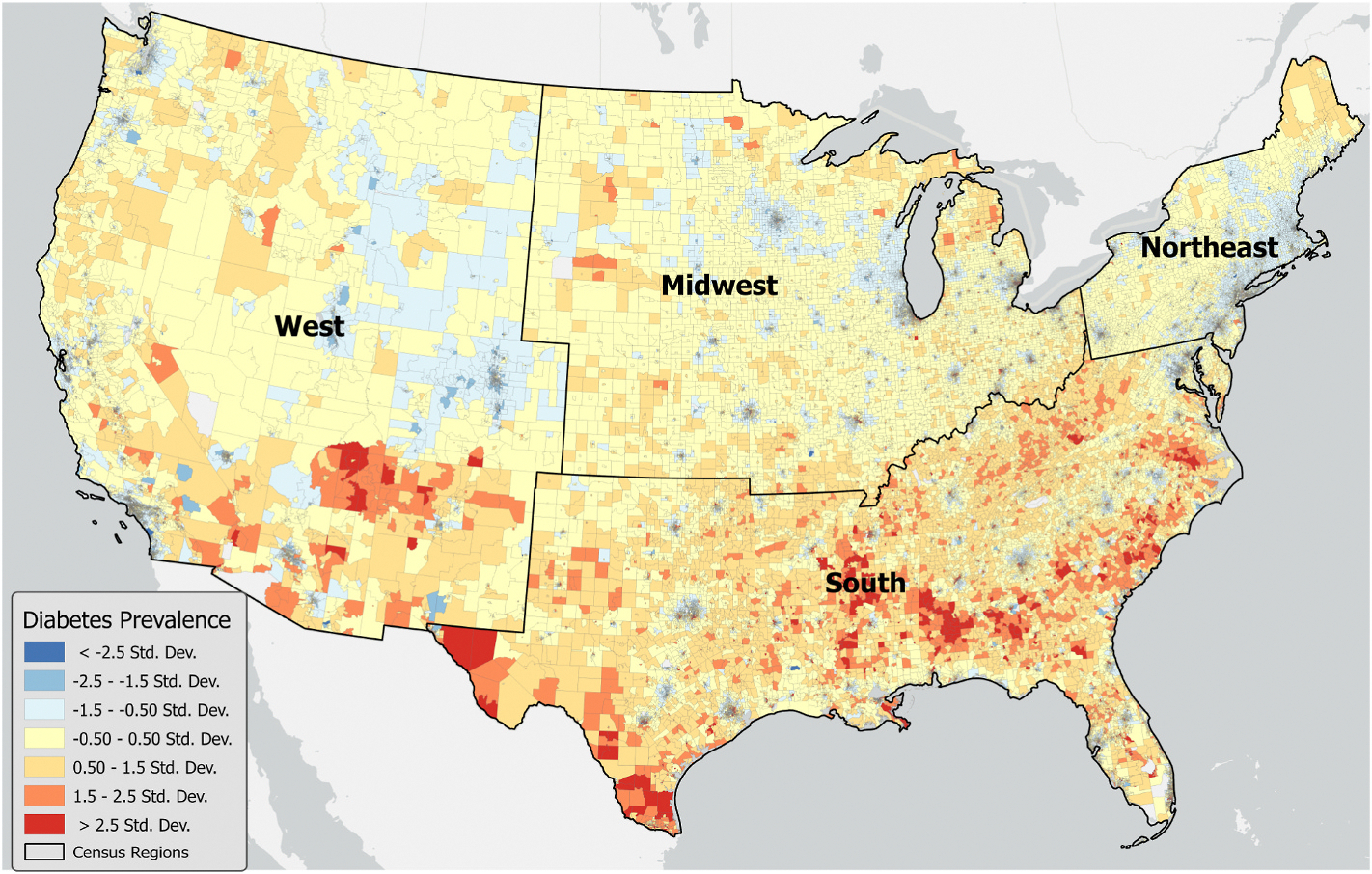
Prevalence of 2019 model-based estimated diagnosed diabetes at census tracts in the contiguous US, with the boundaries of census regions. Colors indicate 7-class standard deviations compared to mean value. (For interpretation of the references to color in this figure legend, the reader is referred to the Web version of this article.)

The model-based age-adjusted estimates of the prevalence of diagnosed diabetes among adults was extracted from the 2021 PLACES release using 2010 census tract geography. The 2021 PLACES release^1^ uses model-based estimates from the Behavioral Risk Factor Surveillance System (BRFSS) 2019 data, the Census Bureau 2019 county population estimate data, and the American Community Survey (ACS) 2015–2019 estimates. Initiated by US Centers for Disease Control and Prevention’s (CDC) Division of Population Health, PLACES provides health and health-related data using small-area estimation for various geographical aggregations across the United States. A full description of PLACES methodology can be found elsewhere (Centers for Disease Control and Prevention, 2024b; Zhang et al., 2014). This study utilized the age-adjusted, model-based estimated measure of the “diagnosed diabetes among adults ≥18”. Briefly, a multi-level regression model and post-stratification approach was applied to the Behavioral Risk Factor Surveillance System (BRFSS) survey and American Community Survey (ACS) data to compute the probability among adults with model-based estimated diagnosed diabetes. The probability was then applied to the population estimates for 2010 census tracts, generating a model-based estimated prevalence. The spatial distribution of model-based estimated diagnosed diabetes prevalence is shown in Fig. 1. We acknowledge that these are not direct measures and, for simplicity, use the term *diabetes prevalence* throughout the article to refer to the prevalence of model-based estimates of diagnosed diabetes.

### 2.2. Racial and ethnic diversity

Racial and ethnic diversity were assessed by the mix of six races and ethnicities at the 2010 census tract geography using data of the 1990, 2000, 2010, and 2020 decennial Censuses from the National Historical Geographic Information System (NHGIS) (Manson et al., 2023). NHGIS implements the geographically standardized methods to provide data from multiple times for a single census’s geographic units. Complete documentation on time series geographic standardization method is available at: https://www.nhgis.org/time-series-tables#standardization. The six racial/ethnic categories include (a) Not Hispanic or Latino – White (single race), (b) Not Hispanic or Latino – Black or African American (single race), (c) Not Hispanic or Latino – American Indian and Alaska Native (single race), (d) Not Hispanic or Latino – Asian and Pacific Islander (single race), (e) the combination of two categories: (1) Not Hispanic or Latino – Some Other Race (single race), and (2) Not Hispanic or Latino – Two or More Races, and (f) Hispanic or Latino, any race. Although the Asian and Pacific Islander populations were separated since the 2000 decennial Census, they remain combined in the NHGIS standardized data; accordingly, they were analyzed as a single group in this study for all subsequent years. For each timepoint, two diversity indexes were calculated: standardized entropy (*EStd*) and Hill’s number (*Hill*). Entropy (*E*) measures population heterogeneity from the six racial/ethical categories, which is then standardized (*EStd*) via dividing by the maximum entropy resulting in values between 0 and 1. An *EStd* of 0 indicates that the area is dominated by one racial group, whereas *EStd* of 1 means that all racial groups have an equal share in the census tract. Hill’s number (Hill, 1973) is used in landscape ecology as a species diversity measure and is adapted by this study as a racial and ethnic diversity measure. It is calculated as N_H_ = *a*^*E*^ where *a* is based on the logarithm used to calculate entropy (*E*) and *E* is the corresponding entropy value at each census tract. Hill’s number is interpreted as the effective number of racial/ethnic groups with a significant share residing in a census tract. While standardized entropy is a commonly used method to assess the level of diversity, the Hill number provides such information in more intuitive terms, that are easy to interpret.

### 2.3. Control variables

Analysis of the association between racial and ethnic diversity trajectories and diabetes prevalence controlled for age (median age), sex (percent female), poverty (percent poverty), marital status (percent married), and public insurance (percent any public insurance) at the census tract level, from the American Community Survey (ACS) 2015–2019 5-year estimates at the 2010 census tract geography. These neighborhood covariates were selected because they are associated with diabetes (Gaskin et al., 2014; Hill-Briggs et al., 2020). It is important to note that the PLACES poststratification estimation process for calculating diabetes prevalence estimate also employs age and sex in its calculations. However, as these are not variables of focus, but important to control for when modeling neighborhood-level diabetes outcomes, we also include these variables as covariates (Kong and Zhang, 2020).

### 2.4. Statistical analysis

#### 2.4.1. Identifying racial and ethnic diversity trajectory clusters

For each census tract, the two calculated diversity indexes (*EStd* and *Hill*) were converted to a corresponding time series of racial and ethnic diversity trajectory over four timepoints (*TS_EStd* and *TS_Hill*). Using the Euclidian distance among all trajectories as the dissimilarity metric, *K*-means clustering was applied to assign census tracts into trajectory clusters based on the change patterns of diversity from 1990 to 2020. The total number of trajectory clusters was decided via the Silhouette score that evaluated clustering results by measuring within-cluster similarity and maximizing dissimilarity from other clusters. Higher Silhouette scores are desirable and indicate more consistent and better separation from the *K*-means clustering results. Census tracts were mapped to compare the spatial distributions of the racial and ethnic diversity trajectory clusters. Changes of racial and ethnic population percentages in census tract over time were visualized by trajectory clusters. For descriptive analysis of trajectory clusters, covariates and diabetes prevalence were summarized and tested with ANOVA.

#### 2.4.2. Global models

Linear mixed models (LMM) were used to investigate the global relationship between diversity trajectory clusters and diabetes prevalence. The baseline LMM used all covariates and trajectory clusters as fixed effects. Two subsequent models accounted for variability in the intercept within geographic levels (states and counties) including: (a) fixed effect with state as the random intercept, and (b) fixed effect with county nested in state random intercept. All three models controlled for median age, percent female, poverty, marital status, and public insurance at the CT level. Model explainability was evaluated using adjusted R-squared (for Model 1) and adjusted conditional R-squared (for Model 2 and Model 3). The residuals of the LMMs were examined by global and local Moran’s *I* to assess spatial dependence and autocorrelation, respectively.

#### 2.4.3. Spatial models

The spatial distribution of diagnosed diabetes prevalence is not random across the contiguous US (Fig. 1) indicating a need to test for the spatial heterogeneity in the relationship between racial diversity trajectories and diabetes. Our primary interest is how modeled associations vary spatially instead of analyzing spatial spillover or regional diffusion patterns. Geographically weighted regression (GWR) estimates local regression models between the dependent and explanatory variables for each neighborhood, defined as the subsets of the observations surrounding a given location. The general GWR equation is defined as:

$$y_{i}=\beta_{0}\left(u_{i},v_{i}\right)+\sum_{k=1}^{m}\beta_{k}\left(u_{i},v_{i}\right)x_{ik}+\epsilon_{i}$$

where $y_{i}$ represents the observed value of the dependent variable at location i. ($u_{i},v_{i}$) represents the geographic location of location i and is where the local regression model is performed. $x_{ik}$ is the value of the k-th explanatory variable at location i; and m represents the total number of explanatory variables. $\beta_{0}\left(u_{i},v_{i}\right)$ is the intercept term for the local regression model at location i, representing the predicted value of $y_{i}$ when all explanatory variables at location $i\left(x_{ik}\right)$ are zero or at their reference category. The value of $\beta_{0}\left(u_{i},v_{i}\right)$ varies according to the location. $\beta_{k}\left(u_{i},v_{i}\right)$ is the regression coefficient for the k-th explanatory variable ($x_{ik}$) at location i, representing the local relationship between the k-th explanatory variable and the dependent variable at location i. This coefficient varies with locations and indicates how the relationship between the variables changes spatially. $\epsilon_{i}$ is the error term for the regression at location i, representing the difference between the observed and predicted values of $y_{i}$.

The size of census tract areas varies substantially in the US, so we defined the neighborhood for local regression models using the number of neighbors instead of a fixed distance bandwidth. The number of neighbors was optimized for the highest corrected Akaike Information Criterion (AICc) value from the fitted GWR models. For the base model (GWR Model 1), only covariates were included. To model the trajectory cluster variable in GWR, the model specification would require dummy-encoding. However, this may lead to potential local multicollinearity and instability issues in GWR models, due to the spatial clustering of the dummy variables (i.e., because of the clustering of trajectory groups, the are many cases where all the dummy variables in neighborhood will be 0). This lack of variance is problematic to estimate variable coefficients. To address this issue, the racial and ethnic diversity trajectories were represented by two numeric values: (1) the slope of the diversity trajectories, derived from the fitted linear regression line over the four timepoints (referred as **slope**), and (2) the diversity index from the most recent timepoints (referred as **latest**). GWR Model 2 included **slope** of the diversity trajectory, which examines if diversity changes over time contributes to the spatially varying relationships. In GWR Model 3, both **slope** and **latest** were included as independent variables to assess simultaneous impacts of trajectory and current diversity on diabetes prevalence. The Box Cox transformation was applied to diabetes prevalence to ensure the dependent variable was approximately normally distributed in the GWR models. The same set of control variables (age, sex, poverty, marital status, and public insurance) were included as independent variables. Model explainability was evaluated using adjusted R-squared and the local significance of individual explanatory variables was defined by Pseudo-t Statistics. GWR model residuals were assessed for spatial autocorrelation using global Moran’s *I*. Census tracts that were identified with a negative association between diversity trajectory (slope) and diabetes prevenance were further examined, and patterns were compared at the census region level. The comparison was also broken down by the urban status of the region (defined by the census Metropolitan Statistical Area (MSA) definition) and by the diversity trajectory clusters.

## 3. Results

### 3.1. Patterns in racial and ethnic diversity trajectories in the US

**Fig. 2.**
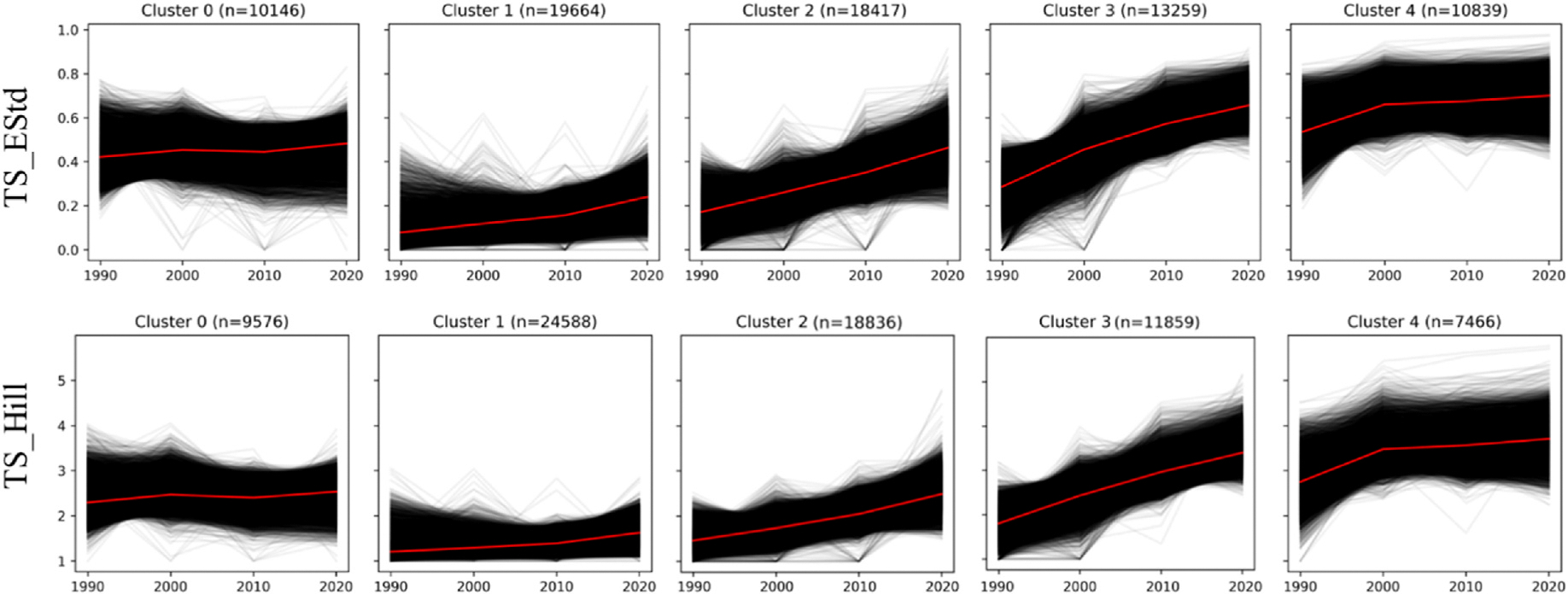
Racial and ethnic diversity trajectory clusters, red lines indicate the median trajectories of each group. (For interpretation of the references to color in this figure legend, the reader is referred to the Web version of this article.)

The optimal number of clusters to categorize racial and ethnic diversity trajectories was 5, based on the evaluation of clustering the *EStd* and *Hill* metrics among all trajectories using the Silhouette score (Supplementary Fig. S1). Five trajectory clusters for each measure were generated by *K*-means clustering of CT-level diversity trajectories over time (Fig. 2). In general, clustering results with the time series of *EStd* and *Hill* were similar. The reference (cluster 1) was characterized by the lowest diversity and little change in diversity over time. Other clusters had moderate diversity with flat changes (cluster 0), moderate 2020 diversity with larger increase over time (cluster 2), higher 2020 diversity with the largest increase over time (cluster 3), and the highest 2020 diversity with a moderate increase over time (cluster 4).

**Fig. 3.**
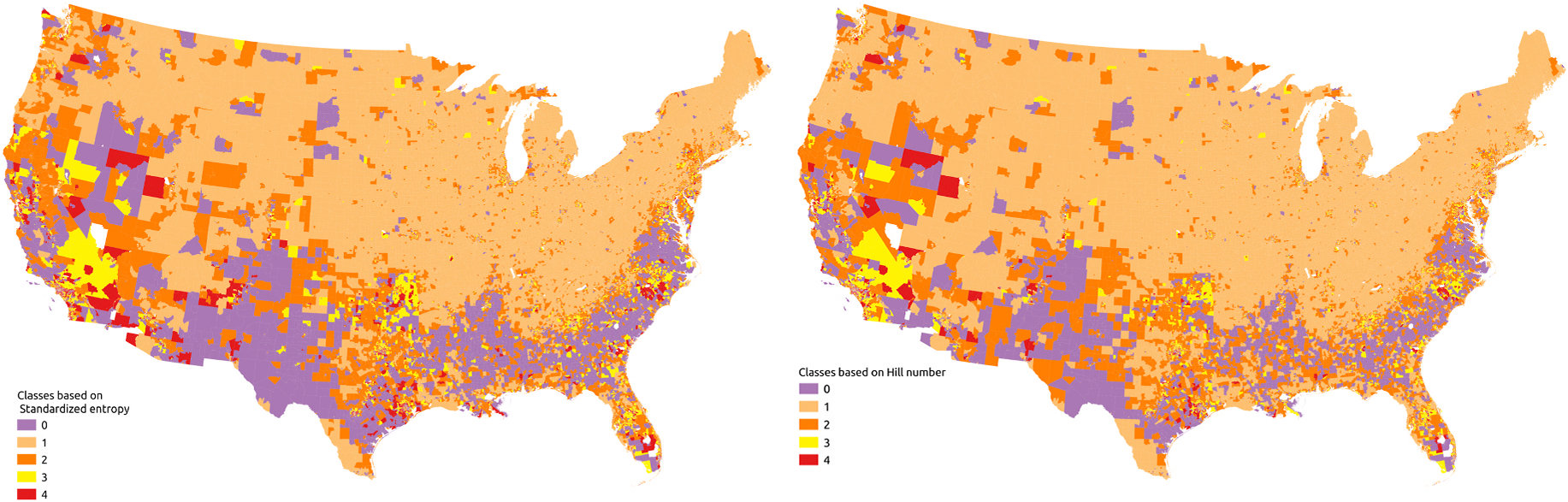
Spatial distribution of racial and ethnic diversity trajectory clusters defined by the time series of standardized entropy (*TS_EStd*, left) and by Hill’s number (*TS_Hill*, right).

**Fig. 4.**
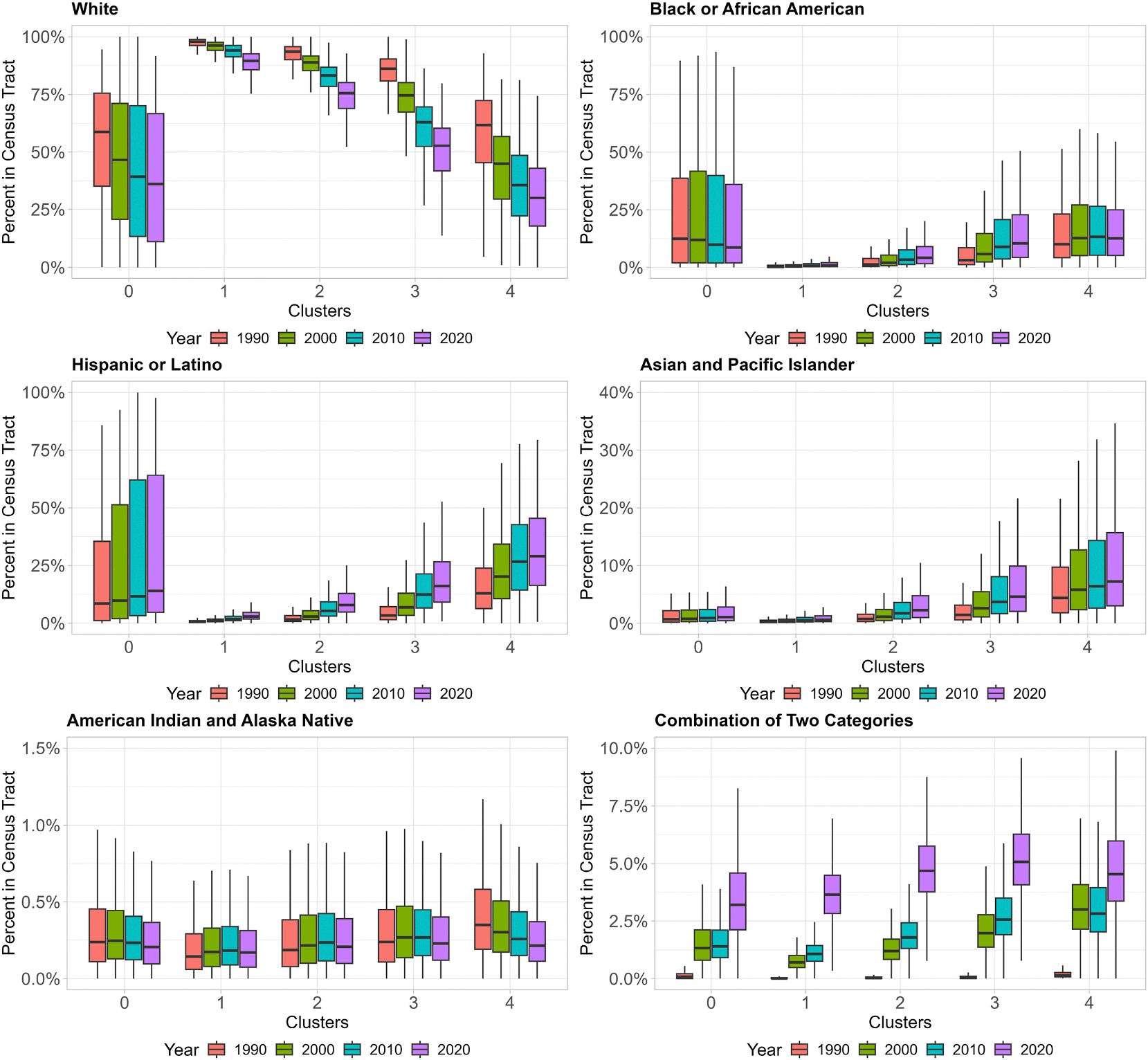
Box plots of changes in the six (Whites, Blacks, Hispanics, Asians, American Indian and Alaska Native, combination of two categories) racial and ethnic population percentages in the diversity trajectory clusters (0, 1, 2, 3, 4) over four timepoints (1990, 2000, 2010, 2020).

The spatial distribution of racial and ethnic diversity trajectory clusters is shown in Fig. 3. Standardized entropy (*EStd*) and the Hill measure (*Hill*) are both transformations of entropy (*E*), which measures the ethnic and racial heterogeneity of a population and thus provides similar information. This similarity is reflected in the clustering and spatial patterns (Figs. 2 and 3), thus from this point on, the focus will be on results of the standardized entropy (*EStd*) metric, whereas the results of *Hill* are included in the supplementary document for comparison and references. Changes in racial and ethnic population percentages in census tract over time were analyzed and compared among trajectory clusters (Fig. 4 and Supplementary Fig. S2). Combining results of both spatial distribution and demographic changes over time, census tracts in the reference cluster 1 are mostly distributed at rural interior and dominated by one racial group (White). Census tracts in cluster 0 are characterized by low to moderate diversity and flat changes over time and are mainly a mix of two racial groups, Black/White in the South-East and Hispanic/White in South/South-West with the proportion of Hispanic population increasing. Cluster 2 contains areas characterized by monoracial (mainly White) populations in the 1990s that became more diverse in the 2000s as shares of other racial/ethnic groups increased continuing into 2010. Census tracts in cluster 2 are located in suburban areas of different metropolitan statistical areas (MSA) and at the boundaries of cluster 0. Cluster 3 contains biracial census tracts in the 1990s that became multiracial in the 2000s, likely due to the increase of Hispanic population and in some cases Asians. Regions in cluster 4 had already been multiracially diverse in the 1990s and continued to increase diversity over the years, however because they already had a high diversity to begin with their increase is not as marked as clusters 2 and 3. Some census tracts in cluster 4 reached *EStd* numbers of 0.8 and higher after year 2000 (Fig. 2), indicating significant shares of White, Black, Asian, and Hispanic population with little opportunity to further increase diversity. Percents of American Indian and Alaska Natives were low throughout all timepoints for all clusters, while percent of population identifying as two or more racial/ethnic categories saw increases across all years for all clusters. For better referencing the clusters, the clusters are assigned with representative labels (Table 1) to highlight their main diversity characteristics and use throughout the rest of this article.

Summaries of covariates by the 5 diversity trajectory clusters are illustrated in Table 2 (by *TS_EStd*) and Supplementary Table S1 (by *TS_Hill*). The results from ANOVA (all p-values <0.001) indicate that the differences observed across the diversity trajectories clusters are statistically significant. Reference cluster 1: Low-Div has the most census tracts (19,601) and is characterized by the oldest population (44 years old, SD = 8), the highest marriage rates (53%, SD = 13), and relatively low poverty (13%, SD = 11) (Table 2). Cluster 0: Mid-Div had the fewest census tracts (10,083) with the highest poverty (15%, SD = 12) and diabetes prevalence (11%, SD = 3.7), along with a younger population (37 years old, SD = 8) and the lowest marriage rates (41%, SD = 13) (Table 2). Cluster 2: Trans_WBH had lower diabetes prevalence (10.2%, SD = 3.5) and had the lowest poverty (12%, SD = 11) (Table 2). Cluster 3: Trans_W was tied for the lowest public insurance (34%, SD = 13) and the lowest diabetes prevalence (10.1%, SD = 3.1) (Table 2). Cluster 4: High-Div had the third lowest prevalence of diabetes (10.6%, SD = 3.4), and was composed of the youngest population (35 years old, SD = 6), higher poverty (18%, SD = 12), and the lowest marriage rates (40%, SD = 12) (Table 2).

### 3.2. Global relationships between trajectory clusters and diabetes

The LMM with fixed and random effects showed a good model fit in explaining the variance in diabetes prevalence (Table 3). The adjusted R-squared values for Model 1 (0.63) indicate that the trajectory clusters and covariates as fixed effects explained a substantial portion of the variance in diabetes prevalence. However, the adjusted conditional R-squared values for Models 2 (0.76) and Model 3 (0.81) shows that incorporating random effects (state and county) improves the model fit and explanatory power. Table 3 also shows the adjusted associations of the diversity trajectory clusters and prevalence of diabetes at the census tract level. The estimates and confidence intervals change across the three models, indicating that the estimated associations are highly dependent on correct model specification (inclusion of counties nested within states). Model 3 shows the strongest effects and the narrowest confidence intervals. This suggests that accounting for the nested spatial structure of administrative boundaries is crucial for accurate estimation, and that unobserved state- and count-level factors are associated with diabetes prevalence. Cluster 0: Mid-Div has the highest prevalence for diabetes of all the clusters (Table 2), and in Models 1 and 2 keeps a positive association compared to reference cluster 1: Low-Div. However, in Model 3, all clusters have a negative association with diabetes prevalence, compared to reference cluster 1: Low-Div. This negative association is the strongest for cluster 4: High-Div, which is characterized by the highest 2020 diversity with moderate increase over time. LMM results for *TS_Hill* clusters are shown in Supplementary Table S2, showing similar patterns across clusters.

**Fig. 5.**
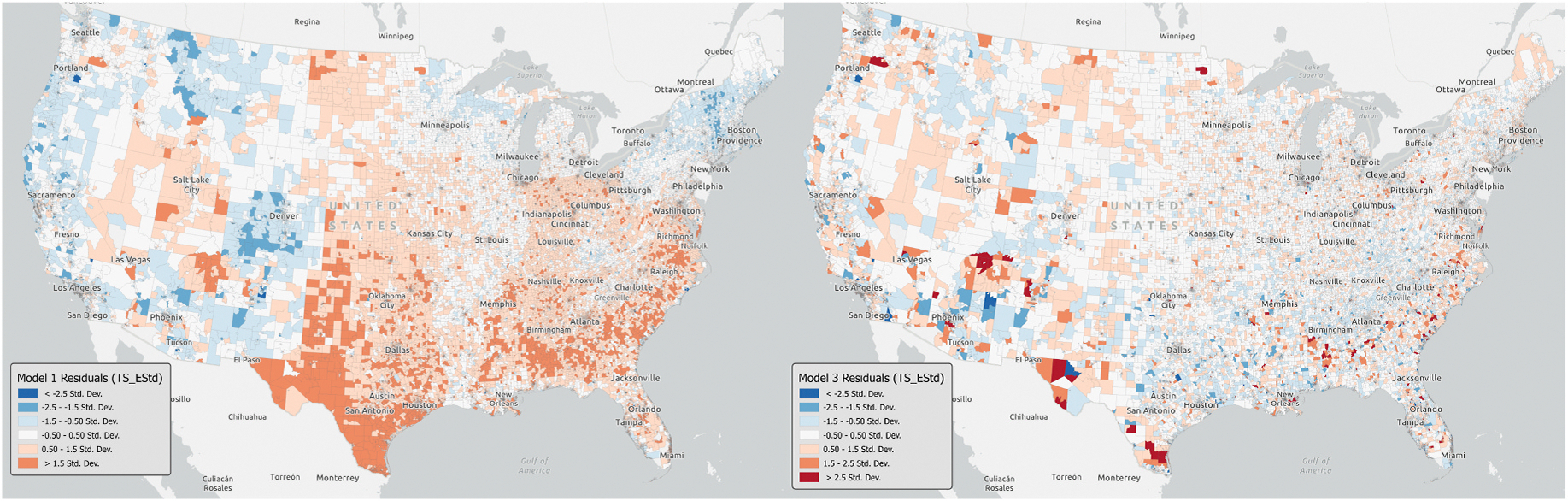
Comparing spatial distribution of linear mixed model residuals. Figure left shows Model 1 residuals (*TS_EStd),* and figure right shows Model 3 residuals. Colors indicating 7-class standard deviations compared to mean value. Model underestimations are shown in red while overestimations are in blue. (For interpretation of the references to color in this figure legend, the reader is referred to the Web version of this article.)

The spatial distributions of standardized model residuals from Model 1 and Model 3 are illustrated in the two maps of Fig. 5, with full results shown in Supplementary Fig. S3. In all the maps, census tract color indicates the 7-class standard deviations of the corresponding standardized model residual when compared to the mean value of all standardized model residuals in the study area. Areas with model underestimations are shown in red colors while overestimations are in blue tones. The map of residuals from Model 1 shows a large portion of census tracts with diabetes prevalence being either underestimated (in red) or overestimated (in blue). The test of spatial autocorrelation in Model 1 residuals using Global Moran’s *I* (z-score = 480.33; p < .0001) confirmed that the over and under-estimations of the prevalence of diabetes are more spatially clustered than would be expected, compared to a random spatial distribution. Full results of the Local Moran’s *I* statistics on model residuals are shown in Supplementary Fig. S4, illustrating the improvements of reducing regions with clustered over/under-estimations when adding state and county-level factors in the LMM models. Models 2 and 3 have improved model fit compared to Model 1, with reduced underestimations in Southern states and reduced overestimations in West coastal states and Northeastern region. However, despite the improvements in explainability after adding state and county-level factors to the LMM models, the spatial autocorrelation in Model 3 residuals remained spatially clustered (Global Moran’s I z-score = 193.07; p < .0001).

### 3.3. Spatial variability in the relationship between racial diversity trajectories and diabetes

For every census tract, the nearest 18 neighboring tracts were used to construct local regression models for the overall GWR model. The neighborhood size selection was based on the golden search selection method that evaluated a series of GWR model performance from 10 to 50 neighbors and finalized with the neighborhood size that granted the lowest corrected Akaike Information Criterion (AICc) value. The GWR model showed good model fit in explaining the variance in diabetes prevalence. The adjusted R-squared values for GWR Model 1 (0.886) indicate that the covariates alone explained a substantial portion of the variance in diabetes prevalence when considering local spatial variability. However, the adjusted R-squared values for GWR Models 2 (0.892) and GWR Model 3 (0.898) shows that incorporating the diversity trajectory (slope) and the most recent diversity index (latest) further improves the model fit and explanatory power. According to the Global Moran’s *I* test, the spatial autocorrelation among three GWR models’ residuals improved from having a spatially clustered patterns in GWR Model 1 (z-score = 13.41; p < .0001) and GWR Model 2 (z-score = 5.92; p < .0001) to randomly distributed in Model 3 (z-score = _−_ 1.33; p = .184). This confirms that the local regression models in GWR Model 3 had effectively accounted for the spatial dependencies in the data and GWR Model 3 is more reliable to explain the spatially varying relationships between diversity trajectory measures and prevalence of diabetes at the census tract level.

### 3.4. Exploring subset of tracts with negative associations between trajectory slope and diabetes

Based on GWR Model 3, a total of 6463 census tracts has a significant negative association between diversity trajectory (slope of *TS_EStd*) and diabetes prevalence. Table 4 illustrates the association between the mean GWR coefficients for covariates and diversity trajectory measures and prevalence of diabetes, stratified by diversity trajectory cluster. GWR coefficients are interpreted in standard deviations; meaning that a value of 0.5 indicates a one-standard-deviation increase in a variable is associated with a 0.5-standard-deviation increase of diabetes prevalence. Across all diversity trajectory clusters, percent of any public insurance has the highest association with diabetes prevalence. Except percent married for cluster 0: Mid-Div, all covariate variables have positive associations with diabetes across all clusters. The latest measure of diversity is also positively associated with diabetes prevalence across all clusters, while diversity slope is negatively associated for all clusters.

A negative association between diversity trajectory and diabetes prevalence can be in the form of a) higher/positive diversity trajectory and lower diabetes prevalence (n = 6100), or b) lower/negative diversity trajectory and higher diabetes prevalence (n = 363). Across all four census regions, the majority of tracts with a significant negative association between diversity trajectory and diabetes prevalence are in urban areas (64.1%). In Table 5, the type of negative association between diversity trajectory and diabetes prevalence is broken out by region and urbanicity. Midwest and Northeast have much higher shares of census tracts from the urban regions, whereas tracts in the South and West states are more evenly from urban and non-urban areas. Urban tracts with a positive diversity trajectory see stronger associations with lower diabetes prevalence in the Midwest and South. For non-urban census tracts, this association is slightly lower in the South. For both urban and non-urban areas, the South contains the most census tracts with significant negative association between diversity trajectory and diabetes, with the majority of these tracts for both urban and non-urban tracts exhibiting a positive diversity trajectory associating with a negative diabetes prevalence.

## 4. Discussion

This study examined the associations between census tract level racial and ethnic diversity trajectories from 1990 to 2020 and model-based estimated diabetes prevalence in 2019. By assessing diversity change over time, we can capture how historical structure of a neighborhood is associated with present day diabetes and glean insight into how racial and ethnic diversity play a role in contextual and compositional structural barriers that impact health outcomes. There are three main findings of this study. First, we found five diversity trajectory clusters across the contiguous US, representing historical diversity trends from 1990 to 2020. Second, global models associating diversity trajectory clusters with diabetes prevalence indicated that compared to census tracts in the reference cluster (which had low overall diversity with little change over time), all other clusters with greater diversity and more increase in diversity over time saw negative associations with diabetes prevalence. Third, spatial models improved model fit and we noted regional patterns of interest for significant negative association between diversity trajectory slope and diabetes prevalence.

The five diversity trajectory clusters were created based on similarity between the 4 timepoints of the racial and ethnic diversity measures, but they are distinct with regards to their socioeconomic characteristics and diabetes prevalence. Clusters 0: Mid-Div and 4: High-Div had higher diabetes rate, coupled with the higher poverty and percent public insurance rate. These clusters were also marked by high diversity in 1990. Trajectory clusters with lower diabetes prevalence tended to have significantly lower poverty (e.g., ~12–14% in clusters 2: Trans_WBH, 3: Trans_W vs. 18%-20% in clusters 0: Mid-Div, 4: High-Div) and higher marriage rates, pointing to greater social and economic stability (Table 2). The reference cluster 1: Low-Div consisted of tracts with persistently low diversity and minimal change over time (Fig. 2), and Fig. 4 illustrates that these low diversity census tracts are predominantly white and remain white. These areas were on average less socioeconomically disadvantaged (poverty ~13%, highest marriage ~53%) but had the second highest diabetes prevalence (11.5). It is interesting to compare this cluster with cluster 0: Mid-Div, with the highest diabetes rate, in Figs. 2 and 4. Many tracts in cluster 0: Mid-Div are likely racially isolated enclaves and considered with cluster 1: Low-Div, tracts from these clusters reflect how low-diversity areas can span both privileged and marginalized communities. The disparities between trajectory clusters in socioeconomic status and health outcomes are consistent with prior studies showing that racially segregated or isolated neighborhoods often face concentrated poverty and higher chronic disease burden (Gaskin et al., 2014; Williams and Collins, 2001). Our findings extend this knowledge by demonstrating that these relationships hold when looking at the history of neighborhood diversity: areas that remained either highly homogeneous with low diversity (cluster 1: Low-Div) or moderately diverse but with little change (cluster 0: Mid-Div) have higher diabetes prevalence today than those that experienced greater racial and ethnic change in diversity.

Our findings also suggest that long-term trajectories of racial composition capture meaningful social realities that differentiate neighborhoods in ways relevant to health. Past research has noted the emergence of different “paths” of racial composition: some communities remain predominantly one race, whereas others experience gradual integration or shifts toward multiethnic makeups (Lichter et al., 2015; Logan and Zhang, 2010). Our cluster analysis empirically confirms such patterns at a national scale. Importantly, we show that these different diversity pathways carry distinct implications for community well-being. Clusters characterized by long-term multiracial integration or increasing diversity (such as clusters 2: Trans_WBH, 3: Trans_W, and 4: High-Div) had significantly lower diabetes prevalence (10.1–10.6%) compared to clusters that remained racially stagnant (Table 2). The implication is that how a neighborhood’s diversity evolves over time may be as important for health as its present-day demographics. Yet, prior studies have rarely examined health outcomes in the context of changing neighborhood diversity. Most segregation–health research uses static, one-time measures of composition or segregation (Yang et al., 2020). By evaluating trajectory groups, our study addresses this gap, providing new insight that the temporal dynamics of racial diversity are linked to chronic disease outcomes. This dynamic perspective is especially pertinent given the rapid growth of Hispanic and Asian populations in recent decades, leading to more multi-ethnic neighborhoods whose health impacts are not well understood (Yang et al., 2020). In Fig. 4, across all clusters, Hispanic/Latinos are making up greater shares of census tracts marking a demographic transition that is likely to have implications for the social and environmental fabrics that can impact health outcomes. This sets the stage for understanding the direct associations between diversity trajectories and diabetes in our models.

The global models assessing racial and ethnic diversity trajectory groups and diabetes prevalence found that census tracts belonging to clusters with high starting diversity or increasing diversity over time were associated with a lower prevalence of diabetes, compared to reference cluster 1: Low-Div with low diversity and little change (Table 3). These results build on previous literature, which did not assess diversity trajectories, but did find that tract level racial and ethnic diversity was associated with a decreased likelihood of metabolic syndrome (one component of which is increased high fasting blood sugar), using data from the 2003 to 2008 National Health and Nutrition Examination Survey (Li et al., 2019). Of note, model performance significantly improved when accounting for county and state nesting, suggesting a strong spatial and potentially administrative component in the relationship between diversity trajectories and diabetes prevalence, potentially tied to historical practices of purposeful neighborhood segregation. Cluster 4: High-Div, characterized by the highest 2020 diversity and a moderate upward trajectory, also exhibited the largest negative association with diabetes prevalence at 0.7% less prevalence than homogenous cluster 1: Low-Div tracts after accounting for socioeconomic differences. These results support our hypothesis that neighborhoods with increasing or sustained racial/ethnic diversity would be associated with lower diabetes prevalence. They also align with broader evidence that reductions in racial residential segregation can yield health benefits, often tied through economic trajectories (Bilal et al., 2019; Chen et al., 2023; Siegel et al., 2024; Xiao et al., 2022).

The associations between residential racial and ethnic diversity trajectory groups and diabetes prevalence were better explained when considering spatial variability. The GWR base model with all covariates showed improved explainability compared to the global models. When adding the two diversity trajectory measures, slope for rate of changes and latest diversity index, GWR Model 3 further improved explainability and were able to effectively account for the spatial dependencies that persisted in the relationship. The relationship between diversity trajectories and diabetes prevalence varies markedly across geographic space. Approximately 6463 census tracts (8.8% nationwide) exhibited a significant negative association between increasing diversity and diabetes prevalence, with the most census tracts in the South. In the South, these tracts were both urban and non-urban with increasing change in diversity while in the Midwest and Northeast, they appeared predominantly in urban settings. This pattern reflects known demographic histories of Southern rural areas with long-standing Black or Latino populations and Midwestern cities undergoing integration and suggests different pathways through which diversity may promote health. Crucially, the diversity trajectory slope was consistently protective, while current diversity levels showed a slight positive association with diabetes, likely reflecting multiethnic urban disadvantage. This distinction highlights that change in diversity, not static composition, may be more beneficial for reductions in diabetes prevalence.

While this study did not assess the specific mechanisms linking diversity trajectories with diabetes prevalence, they are important to discuss. The associations observed between diversity trajectories and diabetes prevalence likely arise from both compositional and contextual mechanisms. Compositional effects may partially explain differences in prevalence, as neighborhoods that remained homogeneous likely include higher proportions of historically marginalized populations (e.g., Black or Latino residents) who experience elevated diabetes risk due to structural disadvantages accumulated over the life course (Gaskin et al., 2014; Hill-Briggs et al., 2020). These neighborhoods may reflect long-standing disinvestment, poor built environments, or reduced access to quality healthcare—features strongly tied to historical redlining and racial segregation (Bailey et al., 2017; Mujahid et al., 2023). By contrast, tracts which experienced the greatest increases in diversity may have undergone structural improvements over time, such as new housing, retail investment, or enhanced medical infrastructure—factors that promote diabetes prevention and management. In some regions, rising diversity could reflect economic revitalization or public investment in historically segregated communities, altering the contextual environment to support healthier living. In other cases, diversity gains may reflect gentrification, which has been linked to improved health resources but also to displacement-related stress (Tulier et al., 2019). These findings are also consistent with evidence that increasing racial integration is associated with reduced allostatic load and cardiometabolic risk, particularly in areas where minority residents gain access to formerly exclusive resources (Kramer and Hogue, 2009; LaVeist et al., 2008). The protective associations seen in clusters with increasing diversity thus likely reflect not only who lives in these areas, but how those areas have changed socially, economically, and structurally over time.

Our approach offers promise in advancing our understanding of the association between residential racial and ethnic diversity and diabetes prevalence; however, there were some limitations. Only the estimated prevalence of diagnosed diabetes was analyzed as the health outcome in this study and thus the total prevalence of diabetes (both diagnosed and undiagnosed) may be underestimated. According to the National Diabetes Statistics Report by CDC, self-awareness of having diabetes remains to be low and accounts for 3.4% of all US adults and 22.8% of all US adults with diabetes. Previous literature had also pointed out that factors including lack of insurance and limited access to health services could lead to lower documented diabetes prevalence (Cowie, 2019; Fisher-Hoch et al., 2015). Information underlining the changes in neighborhood diversity was not explored and could be of interest for future research in relation to the findings of this study. This was an ecological study and did not account for other neighborhood-level factors that may be associated with diabetes prevalence, such as the food environment, social environment, or the built environment (Mujahid et al., 2023). The small-area, model-based diabetes estimates from the CDC PLACES database are the expected diabetes prevalence. Also, the models used to generate the diabetes prevalence used some of the same covariates as this study. Using diabetes diagnoses contained in localized electronic health records to estimate prevalence and associations with neighborhood diversity is a potential direction for future research (Dalton et al., 2022).

## 5. Conclusion

Our study provides new evidence that long-term changes in neighborhood racial/ethnic composition are linked to contemporary disparities in diabetes. Census tracts that remained predominantly white or saw little change in diversity over time had the highest diabetes prevalence, whereas tracts that became more racially diverse over the decades exhibited lower diabetes prevalence. These associations held even after accounting for age, poverty, and other factors, underscoring that a history of integration may independently promote better diabetes outcomes at the neighborhood level. We also demonstrated important spatial nuances: the protective effect of increasing diversity on diabetes was observed nationwide but was strongest in certain regions (e.g., both Southern cities and rural areas, Midwest cities), reflecting America’s varied landscape of segregation and integration. These findings emphasize that neighborhood context mediates the effects of diversity. Regions differ in economic histories, segregation patterns, health policies, and health infrastructure, shaping how integration translates into improved health. Our findings support a growing body of literature suggesting that efforts to reduce residential segregation and support stable, diverse communities could help mitigate chronic disease burdens. Public health practitioners and urban planners should pay special attention to neighborhoods following adverse diversity trajectories – for example, persistently segregated minority enclaves – as these areas may face entrenched health risks and require targeted interventions. Conversely, diversifying neighborhoods appear to experience health gains, which policy can reinforce by ensuring such communities receive adequate resources (e.g., clinics, healthy food outlets) and by protecting against displacement of vulnerable groups. These insights can inform researchers studying health equity by highlighting the importance of life-course exposure to neighborhood contexts (Yang et al., 2020) and equip policymakers with a clearer roadmap of which communities are most in need of structural interventions.

## Supplementary Material

1

## Figures and Tables

**Table 1:** Representation labels for identified clusters of racial and ethnic diversity trajectories in the U.S.

Cluster ID	Cluster Label	Characteristic Highlights

Cluster 0	Mid-Div	Medium diverse areas with significant shares of Blacks and Hispanics minorities
Cluster 1	Low-Div	Predominantly White areas with significant shares of Whites (over 80% of Whites even in 2020)
Cluster 2	Trans_WBH	The transformation from areas dominated by Whites to medium-diverse areas dominated by Hispanics, Blacks, and high-diverse areas
Cluster 3	Trans_W	The transformation from low diversity dominated by Whites to medium diversity dominated by Whites
Cluster 4	High-Div	High-diverse areas, mostly located in urban areas in large cities

**Table 2:** Covariate summaries (means and SD) by diversity trajectories clusters for *TS_EStd.*

			Diversity Trajectories Clusters by TS_EStd					
Characteristic	N	All tracts N = 72,033	0: Mid-Div N = 10,083	1: Low-Div N = 19,601	2: Trans_WBH N = 18,355	3: Trans_W N = 13,221	4: High-Div N = 10,773	p-value^a^

Median age	72,033	40 (8)	37 (8)	44 (8)	41 (7)	37 (6)	35 (6)	<0.001
Percent female	72,033	50.8 (4.3)	50.6 (5.4)	50.9 (3.3)	51.1 (3.8)	51.1 (4.1)	50.0 (5.5)	<0.001
Percent poverty	72,033	15 (12)	20 (12)	13 (11)	12 (11)	14 (11)	18 (12)	<0.001
Percent married	72,033	47 (14)	41 (13)	53 (13)	49 (13)	45 (13)	40 (12)	<0.001
Percent any public insurance	72,033	37 (14)	42 (15)	39 (13)	34 (13)	34 (13)	37 (14)	<0.001
Percent diabetes	72,033	11.0 (3.7)	12.8 (4.1)	11.5 (3.8)	10.2 (3.5)	10.1 (3.1)	10.6 (3.4)	<0.001

a One-way ANOVA.

**Table 3:** Adjusted R-square, adjusted conditional R-square, and associations of the *TS_EStd* diversity trajectory clusters and prevalence of diabetes at the census tract level.

	Model 1: all covariates		Model 2: all covariates + state random intercept		Model 3: all covariates + county nested in state random intercept	
R-squared^a^	0.63		0.76		0.81	
Diversity Trajectory Clusters by TS_EStd	Est	95% CI	Est	95% CI	Est	95% CI

0: Mid-Div	**0.41**	0.35, 0.47	**0.13**	0.08, 0.18	**−0.27**	−0.32, −0.22
1: Low-Div	*ref*		*ref*		*ref*	
2: Trans_WBH	**−0.13**	−0.18, −0.08	**−0.16**	−0.21, −0.12	**−0.37**	−0.42, −0.33
3: Trans_W	0.07**	0.01, 0.13*	**−0.04**	−0.09, 0.00	**−0.43**	−0.49, −0.38
4: High-Div	**−0.49**	−0.54, −0.43	**−0.28**	−0.34, −0.23	**−0.71**	−0.76, −0.65
slope	**−72.59**	−76.92, −68.26	**−77.51**	−81.18, −73.85	**−61.31**	−64.88, −57.75

** Besides Model 1 for group 3 (p = .02), all others p < .0001. Covariates are mean centered and standard deviation scaled: age, sex, poverty, marriage status, and public insurance. Bold coefficient CIs do not include 0.

a Adjusted R-squared is used for Model 1, while adjusted conditional R-squared is used for Models 2 and 3.

**Table 4:** The association of mean GWR variables’ coefficients and prevalence of diabetes, stratified by diversity trajectory clusters (mean coefficient values from GWR Model 3). Analysis is limited to census tracts with a significant negative association between diversity trajectory (slope of *TS_EStd*) and diabetes prevalence (n = 6463).

Diversity Trajectory Cluster	Median age	Percent female	Percent poverty	Percent married	Percent any public insurance	Diversity: Slope	Diversity: Latest

**0: Mid-Div** (*n* = 1273, 12.70%)^a^	0.186	0.032	0.178	−0.019	0.592	−0.256	0.050
**1: Low-Div** (*n* = 1825, 9.31%)	0.174	0.028	0.131	0.011	0.594	−0.263	0.142
**2: Trans_WBH** (*n* = 1598, 8.73%)	0.171	0.026	0.156	0.005	0.586	−0.249	0.124
**3: Trans_W** (*n* = 997, 7.58%)	0.191	0.025	0.170	0.001	0.555	−0.257	0.163
**4: High-Div** (*n* = 770, 7.33%)	0.202	0.019	0.152	0.018	0.597	−0.261	0.127

a *n* is the count of census tracts with a significant negative association between diversity trajectory (slope of *TS_EStd*) and diabetes prevalence, followed by its percentages in the diversity trajectory cluster.

**Table 5:** The association of diversity trajectory (measured as the slope of *TS_EStd*) and prevalence of diabetes stratified by census regions, urbanicity, and positive or negative diversity trajectory slope. Only census tracts from GWR Model 3 with a significant negative association between diversity trajectory (slope of *TS_EStd*) and diabetes prevalence are included (n = 6463). Numbers inside the parentheses are percentages of census tracts in the category.

	Urban		non-Urban	
	Positive Diversity Trajectory	Negative Diversity Trajectory	Positive Diversity Trajectory	Negative Diversity Trajectory

**Midwest** (n = 1381)	−0.276 (71.47%)	−0.258 (2.68%)	−0.271 (25.63%)	−0.203 (0.22%)
**Northeast** (n = 1271)	−0.224 (75.53%)	−0.222 (3.23%)	−0.271 (20.69%)	−0.311 (0.55%)
**South** (n = 2631)	−0.268 (51.08%)	−0.270 (5.02%)	−0.247 (41.77%)	−0.234 (2.13%)
**West** (n = 1180)	−0.232 (48.56%)	−0.301 (5.85%)	−0.277 (44.07%)	−0.319 (1.53%)

## Data Availability

Links to the public data sources used in this study, along with the code snippets, are available at: https://github.com/hdscalecollab/diversity-trajectories.GitHub repository for the Diversity-Trajectories Study (Original data) (GitHub repository)

## Footnotes

1 Estimates for New Jersey are not available for measures based on BRFSS 2019. The state did not collect enough BRFSS data to meet the minimum requirements for inclusion in the 2019 annual aggregate data set. However, the seven measures available in BRFSS 2018 will be carried over for New Jersey.

## Appendix A. Supplementary data

Supplementary data to this article can be found online at https://doi.org/10.1016/j.socscimed.2026.119205.
