# Supplementary material for: Exploring racial and ethnic diversity trajectories and diabetes prevalence in the United States
Source: Soc Sci Med. Author manuscript; Available in PMC 2026 Jul 23. (PMC13394639; doi:10.1016/j.socscimed.2026.119205)
Supplement: 1 [file NIHMS2180128-supplement-1.docx]

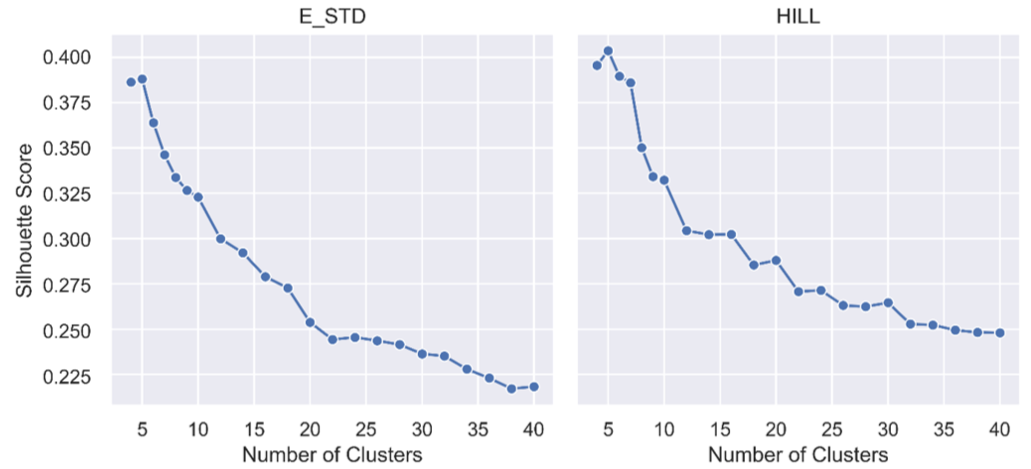


**Figure S1.** Identifying optimal number of clusters for racial and ethnic diversity trajectories: higher Silhouette score is better.


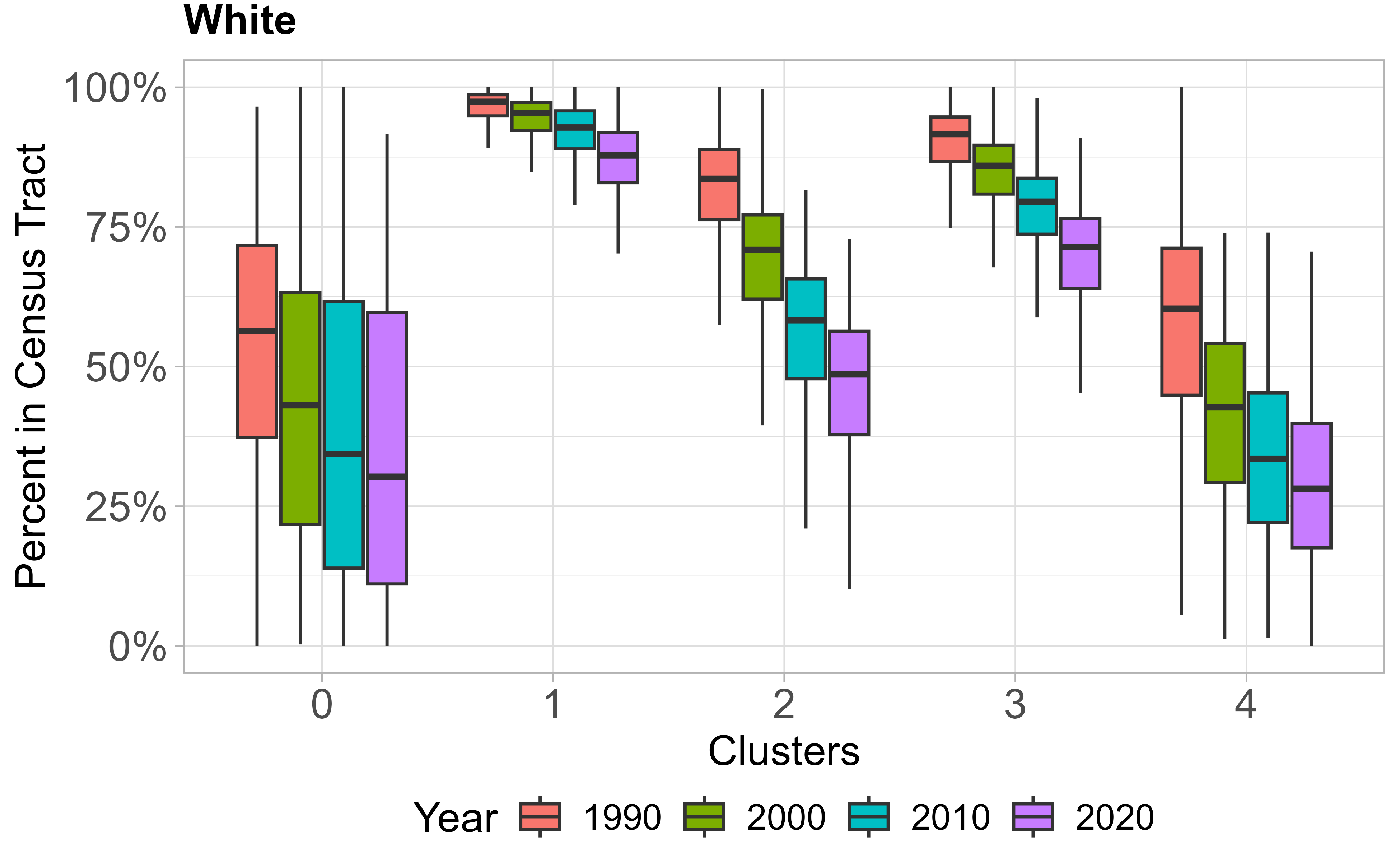

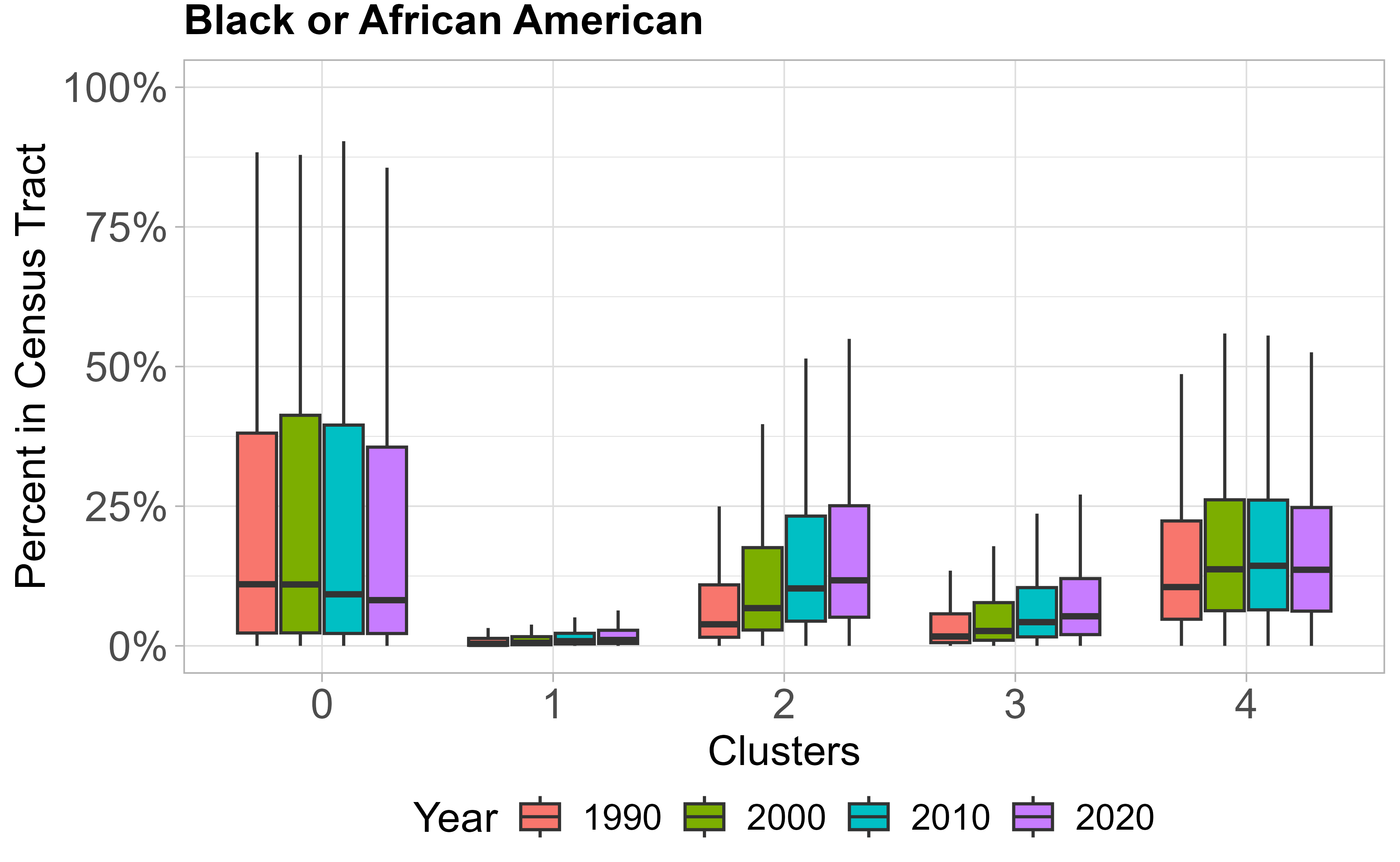

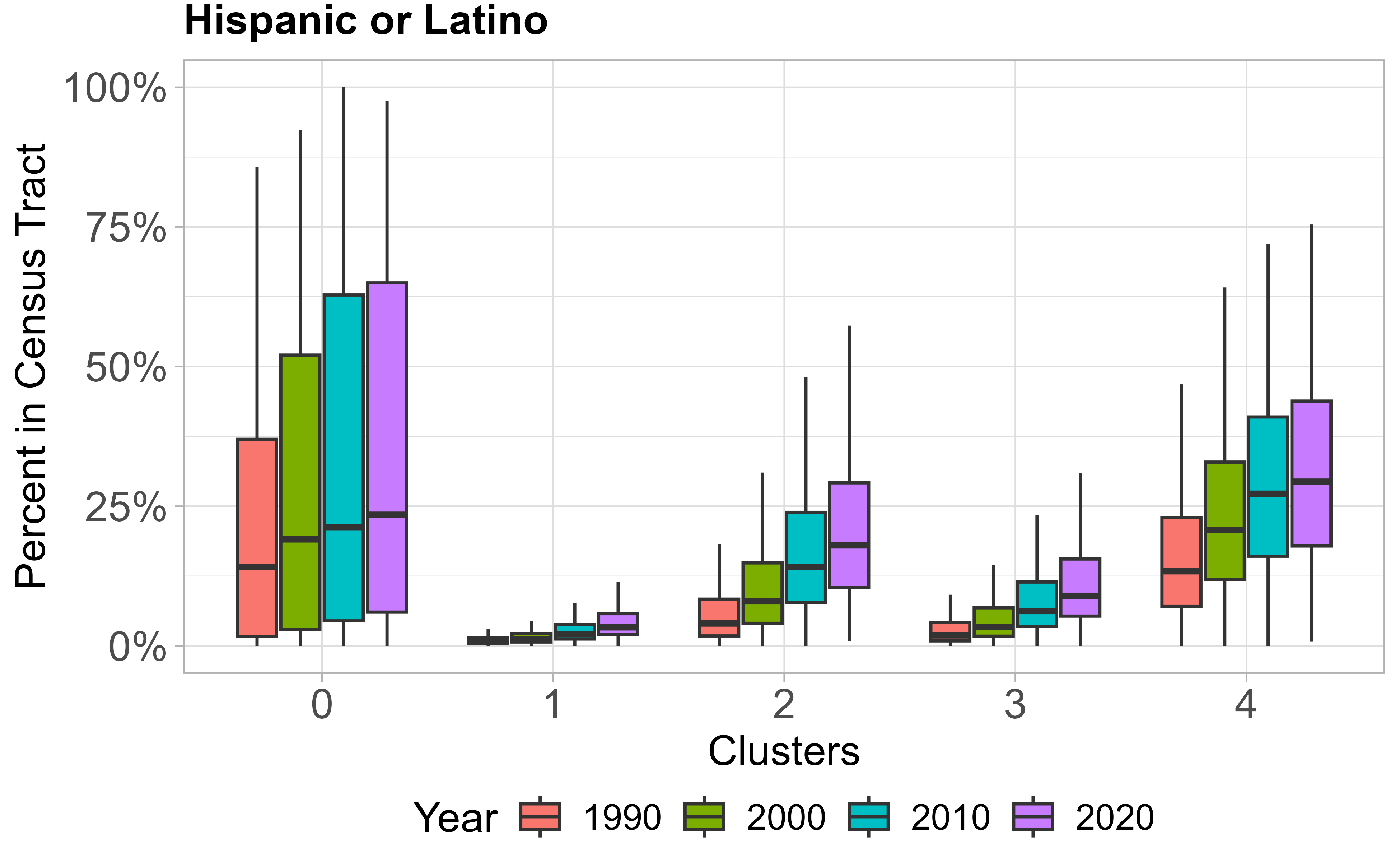

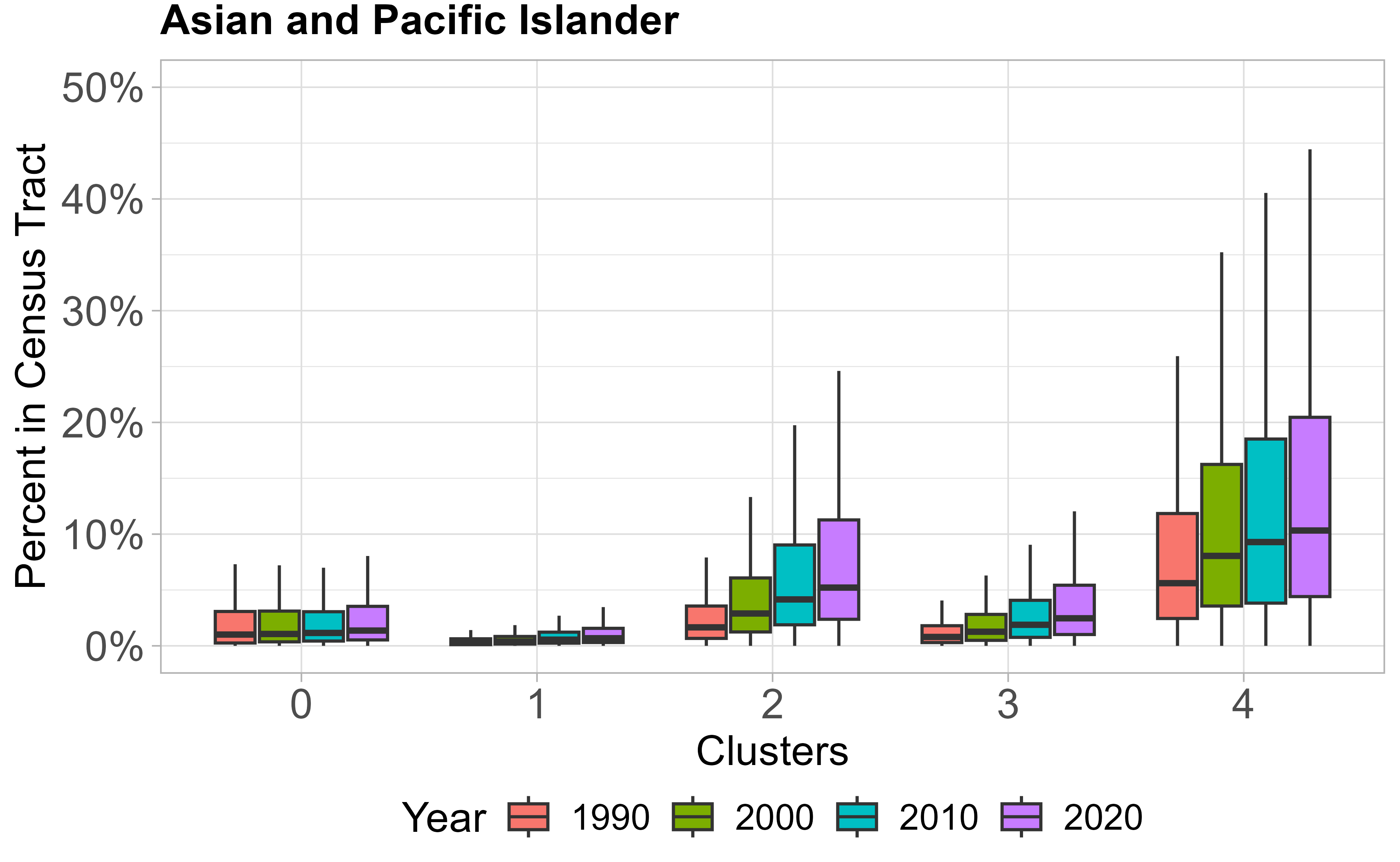

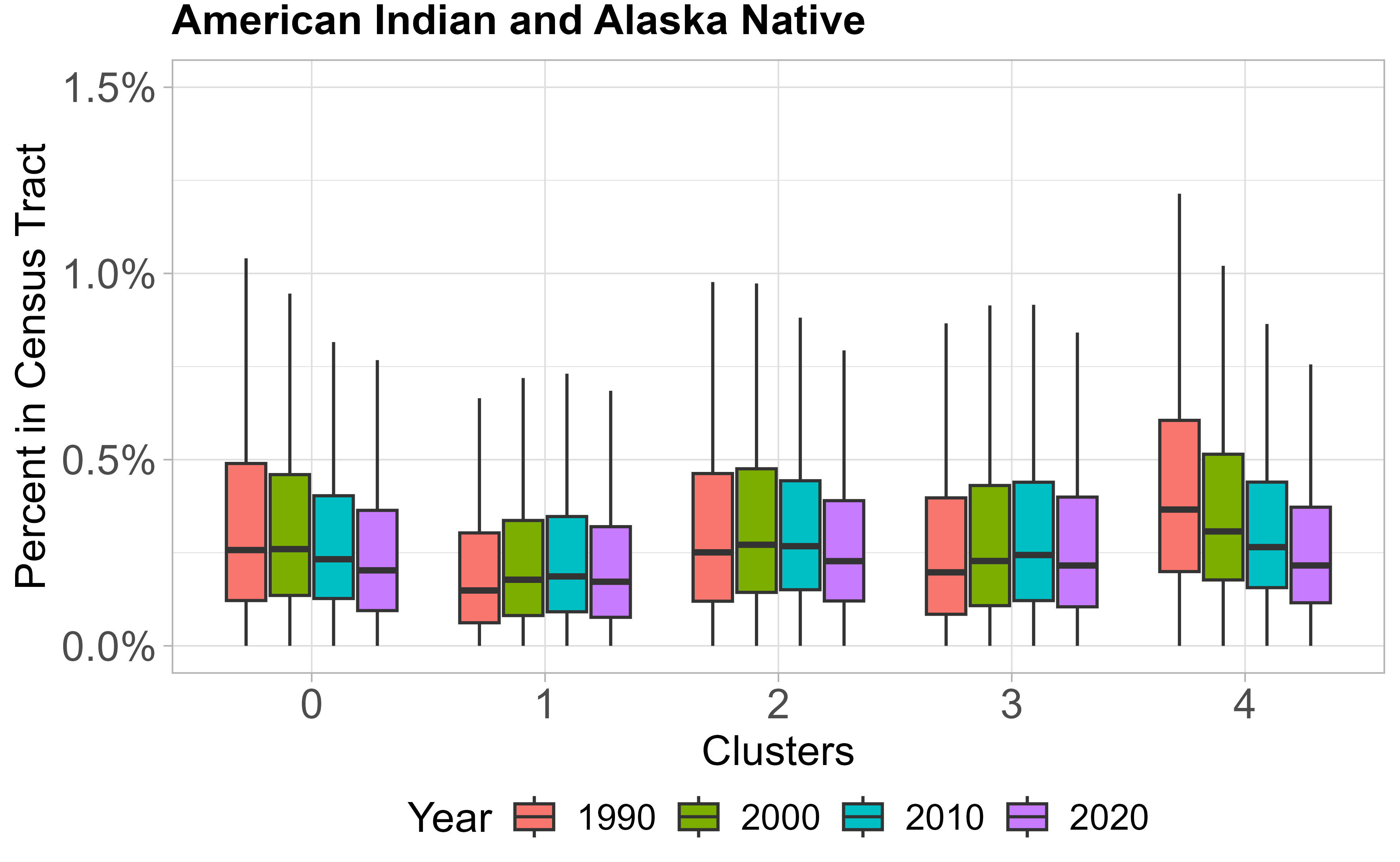

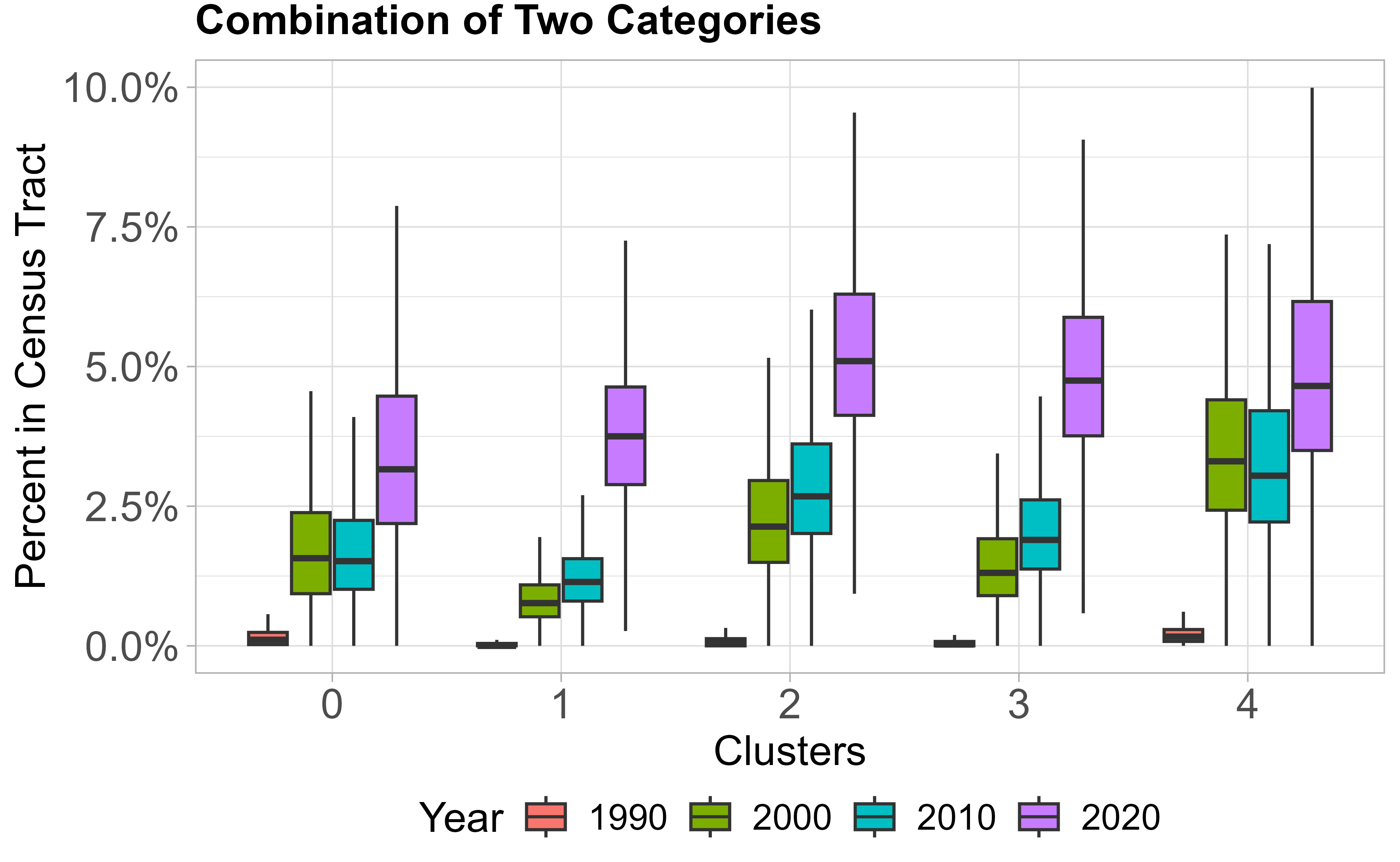


**Figure S2.** Box plot showing the changes in the six (Whites, Blacks, Hispanics, Asians, American Indian and Alaska Native, combination of two categories) racial and ethnic population percentages in Census tract over time. Census tracts were clustered and assigned to one of the racial and ethnic diversity trajectory clusters (0, 1, 2, 3, 4) by the standardized entropy (*TS_Hill*) over four timepoints (1990, 2000, 2010, 2020).

**Table S1.** Covariates summaries (means and SD) by racial and ethnic diversity trajectories clusters for *TS_Hill*

|  |  |  | **TS_Hill** | | | | |  |
| --- | --- | --- | --- | --- | --- | --- | --- | --- |
| **Characteristic** | **N** | **All tracts**  N = 72,033 | **0: Mid-Div**  N = 9,512 | **1: Low-Div**  N = 24,522 | **2: Trans_WBH**  N = 18,760 | **3: Trans_W**  N = 11,812 | **4: High-Div**  N = 7,427 | **p-value^1^** |
| Median age | 72,033 | 40 (8) | 37 (7) | 43 (8) | 40 (7) | 37 (6) | 35 (6) | <0.001 |
| Percent female | 72,033 | 50.8 (4.3) | 50.5 (5.5) | 50.9 (3.3) | 51.1 (4.0) | 51.0 (4.4) | 49.9 (5.6) | <0.001 |
| Percent poverty | 72,033 | 15 (12) | 20 (12) | 13 (11) | 13 (11) | 15 (11) | 18 (12) | <0.001 |
| Percent married | 72,033 | 47 (14) | 41 (12) | 52 (13) | 48 (13) | 44 (13) | 40 (12) | <0.001 |
| Percent any public insurance | 72,033 | 37 (14) | 42 (15) | 39 (13) | 35 (13) | 34 (14) | 37 (14) | <0.001 |
| Percent diabetes | 72,033 | 11.0 (3.7) | 12.6 (3.9) | 11.4 (3.8) | 10.3 (3.5) | 10.1 (3.2) | 10.4 (3.3) | <0.001 |
| ^1^One-way ANOVA | | | | | | | | |

**Table S2.** Adjusted R-square, adjusted conditional R-square, and associations of the *TS_Hill* diversity trajectory clusters and prevalence of diabetes at the census tract level.

|  | **Model 1**: all covariates | | **Model 2**: all covariates + **state random intercept** | | **Model 3**: all covariates + **county nested in state random intercept** | |
| --- | --- | --- | --- | --- | --- | --- |
| **R-squared*** | 0.63 | | 0.76 | | 0.81 | |
| **Diversity Trajectory Clusters by TS_Hill** | Est | 95% CI | Est | 95% CI | Est | 95% CI |
| 0: Mid-Div | **0.47** | 0.42, 0.53 | **0.27** | 0.22, 0.32 | **-0.12** | -0.17, -0.07 |
| 1: Low-Div | *ref* |  | *ref* |  | *ref* |  |
| 2: Trans_WBH | **0.21** | 0.14, 0.27 | **0.13** | 0.07, 0.19 | **-0.24** | -0.30, -0.19 |
| 3: Trans_W | -0.03** | -0.08, 0.02 | **-0.11** | -0.15, -0.06 | **-0.30** | -0.34, -0.26 |
| 4: High-Div | **-0.29** | -0.36, -0.23 | -0.01 | -0.07, 0.05 | **-0.45** | -0.51, -0.39 |
| slope | **-15.65** | -16.72, -14.59 | **-17.22** | -18.12, -16.32 | **-13.69** | -14.55, -12.83 |

*Adjusted R-squared is used for Model 1, while adjusted conditional R-squared is used for Models 2 and 3.

**Besides Model 1 for cluster 3 (p=0.02), all others p<.0001. Covariates are mean centered and standard deviation scaled: age, sex, poverty, marriage status, and public insurance. Bold coefficient CIs do not include 0.


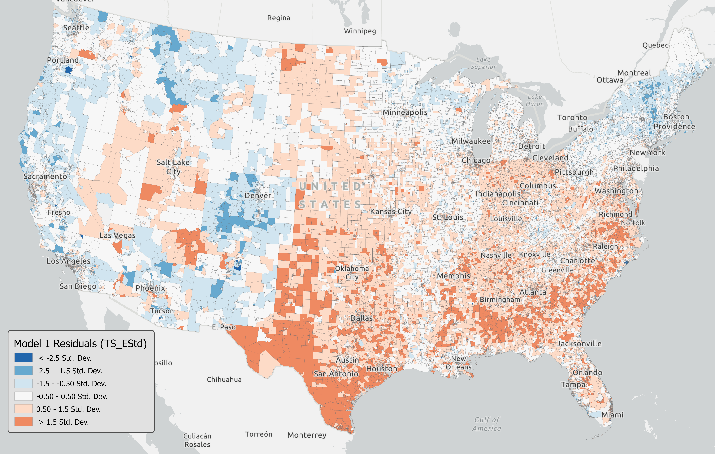

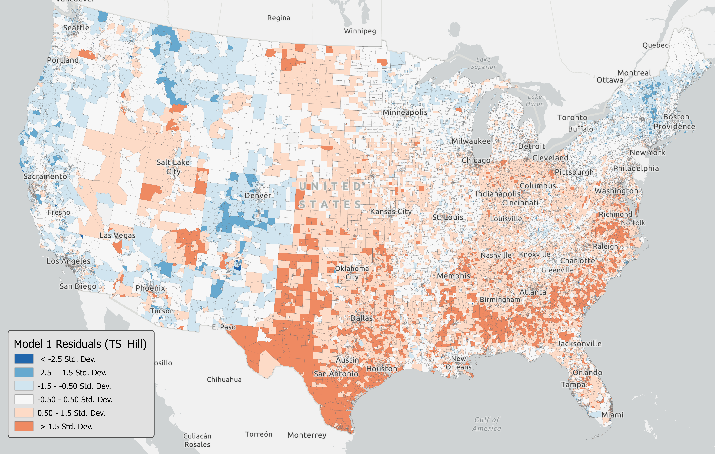
 Model 1: TS_EStd Model 1: TS_Hill


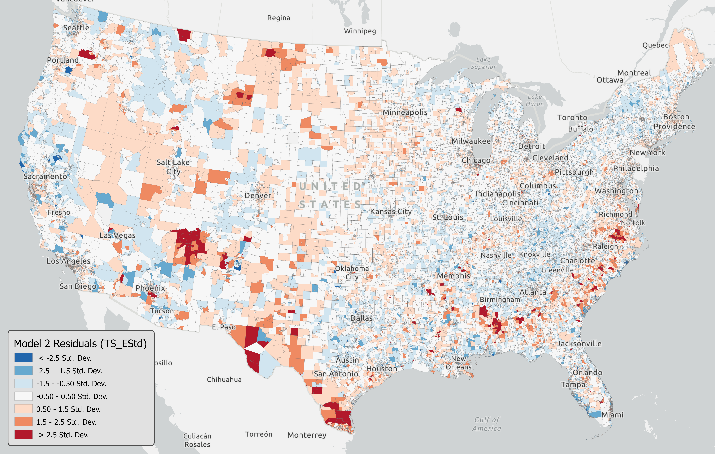

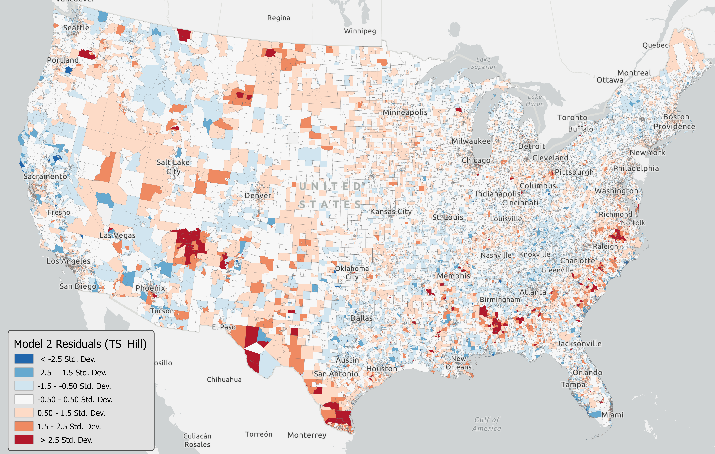
 Model 2: TS_EStd Model 2: TS_Hill


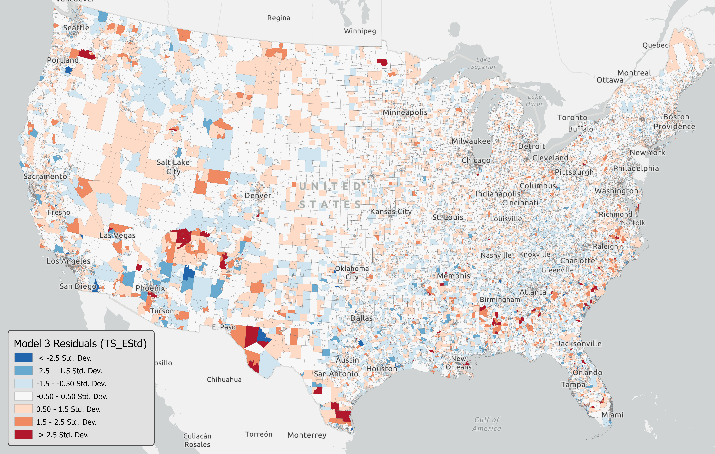

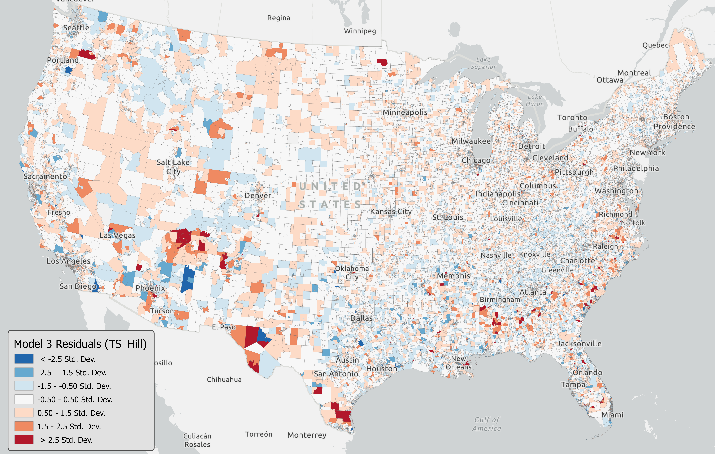
 Model 3: TS_EStd Model 3: TS_Hill

**Figure S3**. Spatial distributions of residuals from linear mixed model with fixed and random effect. Figures left show models with TS_EStd; figures right show models with TS_Hill. From top to bottom are model 1 to model 3 in order. Colors represent the model residuals of Census tracts classified by every 0.5 standard deviation from mean values.


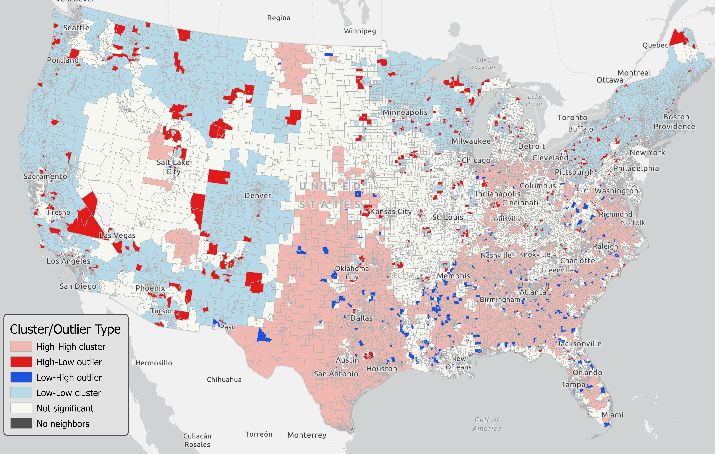

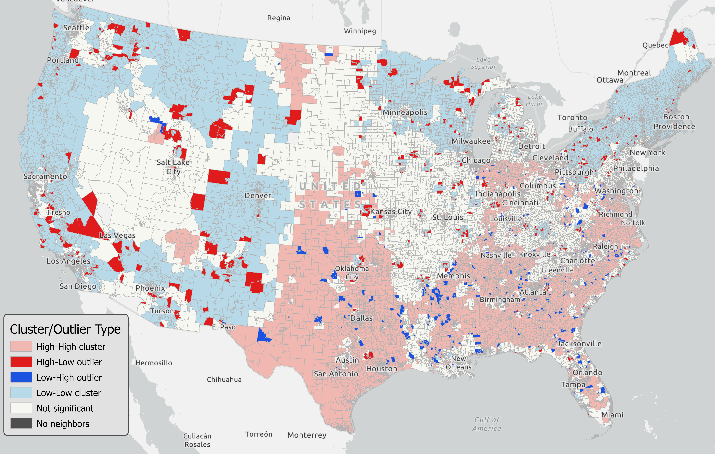
 Model 1: TS_EStd Model 1: TS_Hill


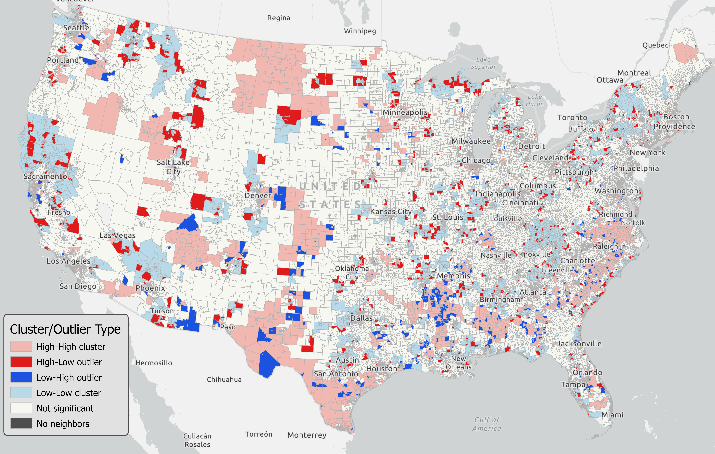

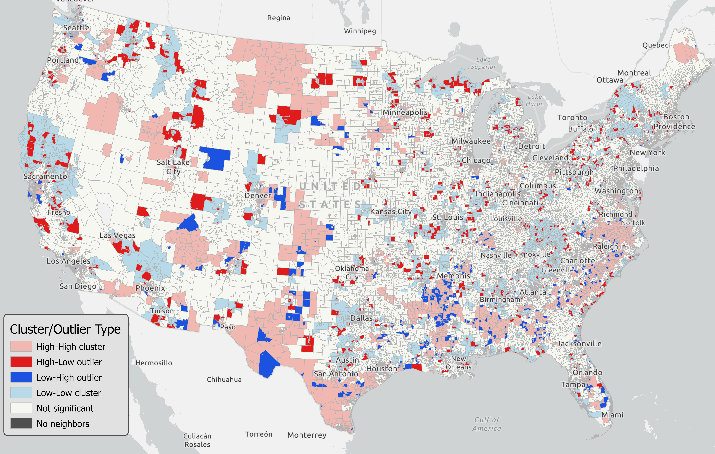
 Model 2: TS_EStd Model 2: TS_Hill


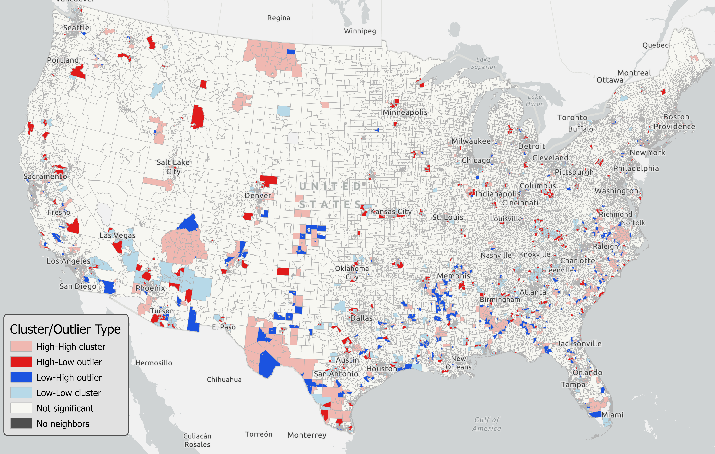

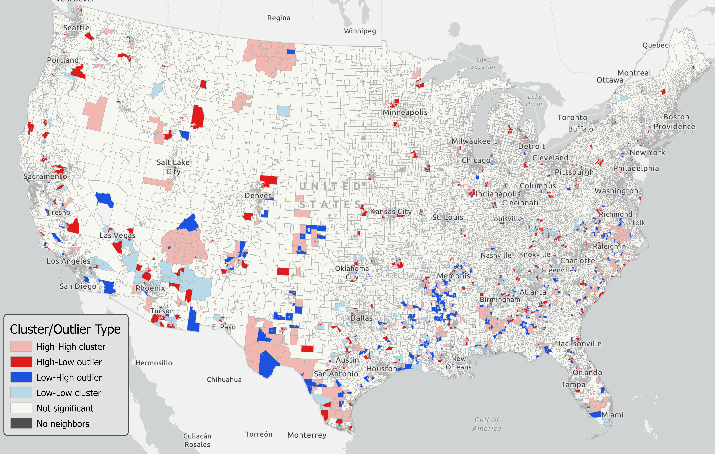
 Model 3: TS_EStd Model 3: TS_Hill

**Figure S4.** Distributions of local clusters/outlier of the residuals from linear mixed models identified by the Local Moran’s I statistics. Figures on the left show models with TS_EStd; figures right show models with TS_Hill. From top to bottom are Model 1 to Model 3 in order. Colors represent identified local clusters and outlier types where Census tracts in light red color indicate a local cluster of overestimated prevalence of diabetes, and light blue color indicate a local cluster of underestimated prevalence of diabetes.
